# Synthetic biology in plants

**DOI:** 10.5511/plantbiotechnology.24.0630b

**Published:** 2024-09-25

**Authors:** Takahiko Hayakawa, Hayato Suzuki, Hiroshi Yamamoto, Nobutaka Mitsuda

**Affiliations:** 1 Mitsubishi Chemical Research Corporation, 16-1 Samon-cho, Sinjuku-ku, Tokyo 106-0017, Japan; 2 Bioproduction Research Institute, National Institute of Advanced Industrial Science and Technology (AIST), Tsukisamu Higashi 2-17-2-1, Toyohira, Sapporo, Hokkaido 062-8517, Japan; 3 Bioproduction Research Institute, National Institute of Advanced Industrial Science and Technology (AIST), Central 6, Higashi 1-1-1, Tsukuba, Ibaraki 305-8566, Japan; 4 Global Zero Emission Research Center, National Institute of Advanced Industrial Science and Technology (AIST), Central 6, Higashi 1-1-1, Tsukuba, Ibaraki 305-8566, Japan

**Keywords:** lignocellulose, luminescent plants, photosynthesis, secondary metabolites, synthetic biology

## Abstract

Synthetic biology, an interdisciplinary field at the intersection of engineering and biology, has garnered considerable attention for its potential applications in plant science. By exploiting engineering principles, synthetic biology enables the redesign and construction of biological systems to manipulate plant traits, metabolic pathways, and responses to environmental stressors. This review explores the evolution and current state of synthetic biology in plants, highlighting key achievements and emerging trends. Synthetic biology offers innovative solutions to longstanding challenges in agriculture and biotechnology for improvement of nutrition and photosynthetic efficiency, useful secondary metabolite production, engineering biosensors, and conferring stress tolerance. Recent advances, such as genome editing technologies, have facilitated precise manipulation of plant genomes, creating new possibilities for crop improvement and sustainable agriculture. Despite its transformative potential, ethical and biosafety considerations underscore the need for responsible deployment of synthetic biology tools in plant research and development. This review provides insights into the burgeoning field of plant synthetic biology, offering a glimpse into its future implications for food security, environmental sustainability, and human health.

## What is “synthetic biology”?

Synthetic biology is a field of science that “applies engineering principles to the natural networks of living organisms to design and build new tools and machines”, enabling creation of artificial biological systems capable of performing new functions, and even new, synthetic forms of life (http://www.futureagriculture.eu/synthetic-biology/synbio/ (Accessed Jul 29, 2024)). Conventional molecular biology adopts a reductive approach to functional analysis by subdividing organisms into individual tissues, cells, molecules, and genes, whereas synthetic biology embraces a constructive approach by designing metabolic pathways as well as genes to produce target substances by utilizing big data, such as genomic information, and creating novel functional cells and biological systems (Aoki et al. 2014; Göpfrich et al. 2018). For example, whereas conventional genetic engineering tends to manipulate the expression of a single gene, synthetic biology aims for greater sophistication, such as the introduction of complete metabolic pathways under strict control, and is characterized not only by the design, synthesis, and expression of DNA components, but also by the incorporation of a biological system’s data and mathematical models based on transcriptomics, proteomics, and metabolomics.

Synthetic biology aims to create new living systems by deliberate design and is distinguished from conventional genetic engineering, i.e., the simple introduction of genes, in that the creation of new systems is “synthetic”. The synthesis of new components intended to be produced by modifying metabolic pathways, such as golden rice, which has been bioengineered to produce high concentrations of β-carotene in the grains (Paine et al. 2005), is one outcome of synthetic biology. The application of genome editing technology to modify metabolic pathways and to create stress-tolerant plants, which is described in the present review, may be a new addition to synthetic biology with regard to deliberate design.

**Figure 1:**
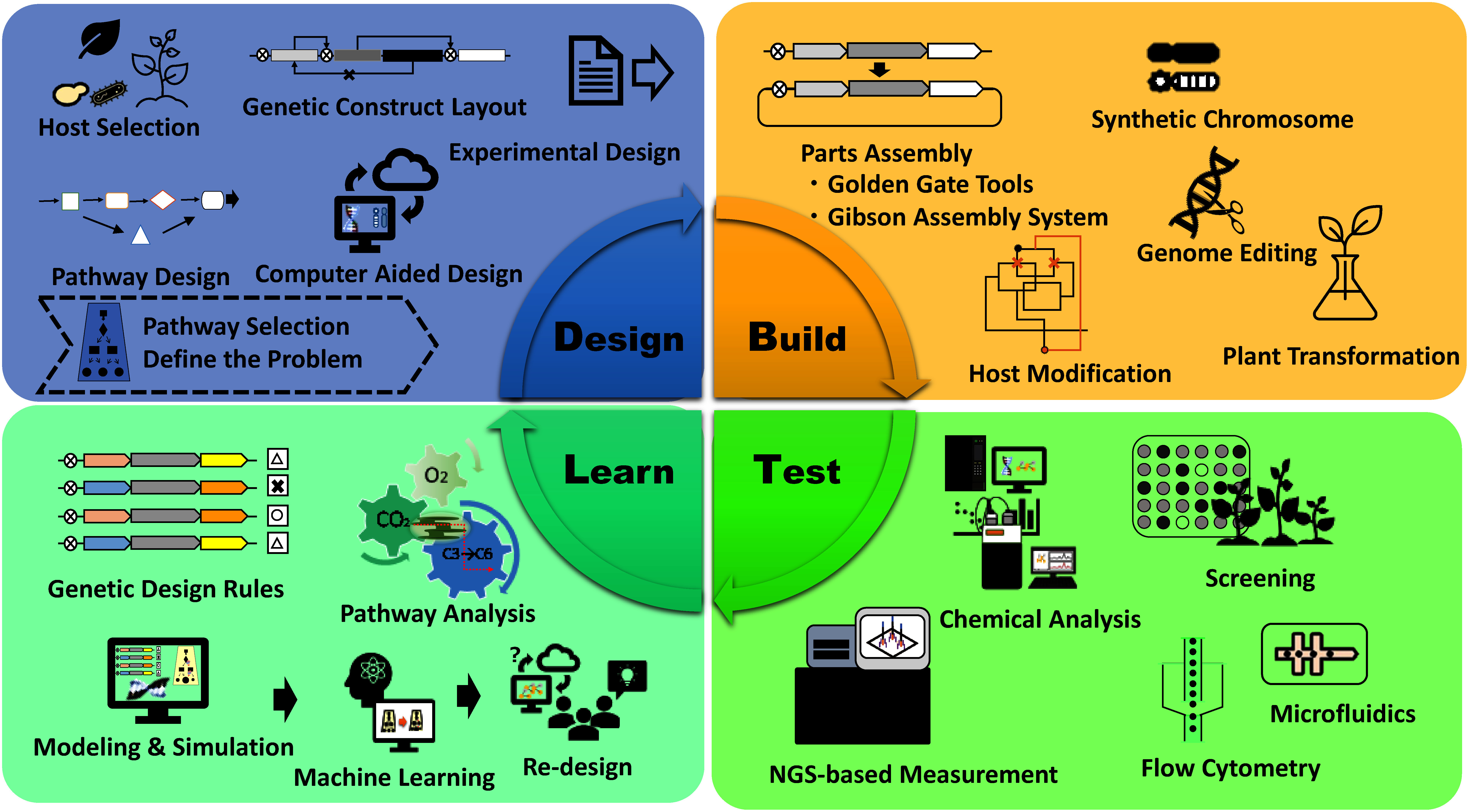
Figure 1. DBTL cycle using various state-of-the-art tools and machines (including plant cases).

In practice, the Design–Build–Test–Learn (DBTL) cycle is used to construct the desired system (Figure 1). By this approach, molecular parts are designed using databases and artificial metabolism design programs (Design); artificial gene clusters are assembled, and the synthesized DNA is incorporated into microorganisms and plants, for example (Build); the productivity is then measured using advanced tools, such as next-generation sequencing instruments and mass spectrometers (Test); and, based on the results obtained, improved measures are found using machine learning with artificial intelligence (AI) and other methods, and the system is redesigned to achieve a given goal (Learn). The cycle is then repeated to achieve the final goal (Gupta et al. 2021; Lv et al. 2021; Shi et al. 2022; Yang et al. 2020). New innovations, such as a million-fold decrease in DNA sequencing costs, a thousand-fold reduction in DNA synthesis costs, the development of genome editing, and the application of AI technologies, have accelerated the DBTL cycle of synthetic biology over the past two decades. The Organisation for Economic Co-operation and Development (OECD)’s Global Forum on Technology in 2023 referred to synthetic biology as “a multidisciplinary area of biotechnology that seeks to harness living systems in research and product development, and the field is already providing breakthrough innovations, e.g., COVID vaccines, and promises others in the areas of health, food security, and the green transition” (https://www.oecd.org/content/dam/oecd/en/networks/global-forum-on-technology/global-forum-on-technology-synthetic-biology-brief-2024.pdf (Accessed Jul 29, 2024)).

## History of synthetic biology

The significant decrease in the cost of DNA synthesis, and the expansion of computer capabilities and associated software (e.g. Nielsen et al. 2016), have played important roles in achieving these breakthroughs. Table 1 lists landmark research achievements of synthetic biology from 2010 to 2024.

**Table 1:** Table 1. Landmark research achievements of synthetic biology from 2010 to 2024 (refer to Meng and Ellis 2020).

Year	Topics	Reference
2010	Synthetic mycoplasma genome	Gibson et al. 2010
	Synchronized bacterial oscillators	Fussenegger 2010
2011	Bacterial growth in stripe patterns	Liu et al. 2011
	**Development of plant sensor**	Antunes et al. 2011
2012	DNA used for data storage	Church et al. 2012
	Whole cell simulation of a mycoplasma	Karr et al. 2012
2013	**Biosynthetic production of artemisinin**	Paddon et al. 2013
	Genomically recoded *E. coli*	Lajoie et al. 2013
2014	Bacteria that sense and record in the gut	Kotula et al. 2014
	Cell-free paper-based sensors and logic	Pardee et al. 2014
	Synthesis of a yeast chromosome	Annaluru et al. 2014
	**EPA/DHA production in plants**	Ruiz-Lopez et al. 2014
2015	*E. coli* dependent on synthetic amino acids	Rovner et al. 2015
	**Biosynthesis of opioids from yeast**	Galanie et al. 2015
2016	In vivo event recorders	Kodandaramaiah et al. 2016
	Synthesis of a reduced mycoplasma genome	Hutchison et al. 2016
	Cello CAD (*1) for *E. coli* logic gates	Nielsen et al. 2016
2017	Synthesis of 5 more yeast chromosomes	Richardson et al. 2017
	CRlSPR-based rapid diagnostics	Gootenberg et al. 2017
	**High accumulation of GABA in tomato**	Nonaka et al. 2017
2018	Self-organizing multicellular structures	Toda et al. 2018
	**High accumulation of astaxanthin in rice**	Zhu et al. 2018
2019	Full synthesis of *E. coli* genome	Fredens et al. 2019
	Carbon fixation in engineered *E. coli*	Gleizer et al. 2019
	Gene circuits with designed proteins	Ng et al. 2019
	**Synthetic production of cannabinoids**	Luo et al. 2019
	**Improving biomass production by introducing photorespiratory bypasses into tobacco chloroplasts**	South et al. 2019
	Development of prime editing in animal cells	Anzalone et al. 2019
2020	**Reconstitution of glycyrrhizin biosynthesis in yeast**	Chung et al. 2020
	**Application of prime editing in plants**	Lin et al. 2020
2021	Metabolic modulation of tumours with engineered bacteria for immunotherapy	Canale et al. 2021
	CO_2_ sequestration sysytem in *E. coli* to achieve theoretical yield of products	Hu et al. 2021
2022	**Semisynthesis of vinblastine from yeast-produced catharanthine and vindoline**	Zhang J et al. 2022
	**Reconstitution of strychnine biosynthesis in tobacco**	Hong et al. 2022
2023	**Commercialization of purple tomato by Norfolk Plant Sciences**	http://www.norfolkplantsciences.com/ (Accessed Jul 29, 2024)
	*E. coli* with swapped genetic code to avoid viral infections and gene transfer	Nyerges et al. 2023
2024	**Development of plants with stronger autoluminescence, commercialization by Light Bio**	Shakhova et al. 2024
	**Reconstitution of vaccine adjuvant QS-21 biosynthesis in tobacco**	Martin et al. 2024

Bold letters represent topics relevant to plant biotechnology development. *1 Cello CAD: Cello is a framework that describes what is essentially a programming language to design computational circuits in living cells (https://www.cidarlab.org/cello (Accessed Jul 29, 2024)).

In 2010, research results by a team at the J. Craig Venter Institute (JCVI) attracted worldwide attention. The team chemically synthesized the entire genome of a species of *Mycoplasma*, a type of bacteria, as a DNA fragment, reconstructed it using the homologous recombination ability of yeast, and introduced the synthesized genome into restriction-minus recipient cells of a closely related *Mycoplasma* species, which subsequently produced proteins based on information from the synthesized genome and initiated self-replicating division and growth as a living bacterium (Gibson et al. 2010). This groundbreaking achievement shows that DNA synthesis and reconstruction can be scaled up to a megabase scale. The results were linked to the development of new energy generation methods, new sensors for environmental monitoring, and the construction of “bacterial factories” for the mass production of pharmaceuticals. Subsequently, in 2016, the team synthesized an artificial genome, reducing the number of genes from 901 to an essential 473 for survival and growth, and successfully introduced the genes into another genome-removed bacterium, which replicated autonomously (Hutchison et al. 2016). Itaya et al. (2018) facilitated the rapid and precise construction of megabase-sized DNA molecules by integrating DNA recombination techniques with conjugation transfer methods for large DNA molecules between *Bacillus subtilis* cells. While conventional transformation methods typically handle DNA molecules of less than 100 kbp, the conjugation transfer system demonstrated the capacity for horizontal transfer of DNA fragments up to 875 kbp by employing *B. subtilis* as a platform for the large DNA assembly. This innovation significantly expedites the assembly process for large DNA molecules. Chin and co-workers created an *Escherichia coli* line with a synthetic genome that was constructed by removing three of the 64 codons in the genetic code (Fredens et al. 2019). This study revealed that the genetic code can be compressed and that bacteria can survive and replicate without certain codes. In the future, these lost codons may be replaced by new sequences encoding non-natural amino acids, creating opportunities to design engineered bacteria that produce non-natural biopolymers.

In eukaryotes, the Synthetic Yeast 2.0 (Sc2.0) consortium, an international project to develop an artificial yeast genome, succeeded in constructing a highly modified and fully functional synthetic version of the baker’s yeast chromosome (Annaluru et al. 2014). This was the first project to design and partially complete a eukaryotic genome from scratch. In 2023, the Sc2.0 consortium succeeded in creating a yeast in which the genome comprises more than 50% synthetic DNA (Bourzac 2023).

## Market for synthetic biology

Synthetic biology has applications in the innovation of food, biofuels, pharmaceuticals, and bioremediation within the academic and life science industry sectors. The synthetic biology market has experienced significant growth over the past decade and is estimated to increase to USD 35.7 billion in 2027, growing at a compound average growth rate (CAGR) of 25.6% during the forecast period (2022–2027) (Synthetic Biology Market—Global Forecast to 2027, MarketsAndMarkets [2023]). This growth will be primarily driven by factors such as the diverse applications of synthetic biology, increased funding for research and development (R&D), declining costs of DNA sequencing and synthesis, and heightened investments in the field. However, concerns regarding biosafety, biosecurity, and ethical implications pose challenges to the market’s growth.The synthetic biology market encompasses various elements, including oligonucleotides, synthetic DNA, enzymes, cloning technology kits, chassis organisms, xeno nucleic acids, and synthetic cells. Among these, oligonucleotides and synthetic DNA accounted for the largest market share (45.9%) in 2021. This dominance is attributed to the growing demand for synthetic DNA, RNA, and genes across diverse applications, such as pharmaceuticals, nutraceuticals, personal care, flavors and fragrances, probiotics, green chemicals, and industrial enzymes.

Segmentation of the market by technology includes gene synthesis, genome engineering, sequencing, bioinformatics, cloning, site-directed mutagenesis, measurement and modeling, microfluidics, and nanotechnology. In 2021, gene synthesis comprised the largest market share (27.7%) in synthetic biology. The genome engineering segment is anticipated to exhibit the highest CAGR of 29.4% during the forecast period (2022–2027). This growth will be fueled by the increasing adoption of engineering technologies for manipulating complex genomes, expanding applications in therapeutics development for cancer and other diseases, and continuing advances in CRISPR toolbox and DNA synthesis technologies.

## Synthetic biology in plants

In recent years, with the development of “synthetic biology” in microorganisms, the application and development of constitutive approaches to material production, nutrient enhancement, biosensors, and stress tolerance, for example, have progressed in plants. Secondary metabolites produced by plants are used in various forms, including pharmaceutical products, spices, dyes, and fragrances. Comprehensive genetic analyses in recent years have elucidated various plant metabolic pathways and, based on these discoveries, the production of plant secondary metabolites by microorganisms using synthetic biology methods has been frequently attempted. In this review, currently topical examples of the application of synthetic biology to plants will be described: modification of plant nutritional components, modification of photosynthetic capacity, production of secondary metabolites (including their production in microorganisms), creation of biosensors, and others, including the latest examples of genome editing. In addition, global trends will be summarized.

## Improvement of nutritional components

### a) Tomato

Tomato is a widely cultivated and consumed vegetable worldwide. The fruits are valued for their exceptional nutritional content, which positions tomato as a functional food. Early adoption of genetic modification (GM) technology in tomato led to the development of the “Flavr Savr™” tomato, heralded as the world’s first commercially available GM crop, which was designed to undergo delayed ripening by suppression of polygalacturonase activity in the pericarp (Bruening and Lyons 2000). While the synthetic biology approach has not been necessarily employed to date, research efforts aimed at enhancing the functional nutritional components of tomato offer insights into manipulation of the metabolic system.

#### a-1) Anthocyanins

Anthocyanins, which are glycosides of anthocyanidins, are plant pigments renowned for their antioxidant properties and contribute to the red, blue, and purple hues in diverse fruits. Certain wild tomato species (*Solanum chilense*, *S. hirsutum*, *S. cheesemanii*, and *S. lycopersicoides*) naturally accumulate anthocyanins in the fruit, resulting in black or purple fruit. Mutant-derived purple tomato varieties include “Purple Smudge”, “V118”, “Sun Black”, “TI00-0028ADBB”, “Purple Cherry”, and “Midnight Roma” (Gonzali and Perata 2020; Gruber 2017; Tilesi et al. 2021). In 2012, Oregon State University released a tomato named “Indigo Rose” with high anthocyanin content in the fruit, which was achieved through cross-breeding with the wild species *S. chilense*, *S. cheesemanii*, and *S. lycopersicoides* (Mes et al. 2008). The tomato “Purple Smudge” features an *Aft-*like gene derived from *S. peruvianum* (https://horticulture.oregonstate.edu/oregon-vegetables/purple_tomato_faq (Accessed Jul 29, 2024)). Li et al. (2011) demonstrated that conventionally bred purple tomato fruit exhibit higher antioxidant activity than red tomato fruit.

In 2008, Butelli et al. enhanced the anthocyanin content in tomato fruit by expressing the Delila (Del) and Rosea (Ros1) transcription factors from snapdragon. These factors encode basic helix-loop-helix and MYB-related transcription factors, respectively. This research revealed a prolonged lifespan of cancer-prone mice fed with purple tomato fruit. Subsequently, Norfolk Plant Science endeavored to commercialize tomato fruit with augmented nutritional properties, resulting in the “Indigo Tomato”, boasting anthocyanin contents twice that of blueberry fruit (http://www.norfolkplantsciences.com/ (Accessed Jul 29, 2024)). The US Department of Agriculture approved this variety for cultivation in September 2022. The US Food and Drug Administration pre-marketing consultation concluded in July 2023 with a “no further questions letter” (https://www.fda.gov/media/170056/download (Accessed Jul 29, 2024)).

The introduction of the MYB12 transcription factor gene, a flavonol-specific activator, into cherry tomato (*Solanum lycopersicum* var. *cerasiforme*) under the control of the fruit-specific *E8* promoter, led to an increase in the flavonol content and antioxidant capacity in the fruit (Wang S et al. 2018).

#### a-2) Carotenoids

Carotenoids are 40-carbon, unsaturated tetraterpenoids consisting of eight isoprene units, and are widely synthesized in plants and animals as fat-soluble natural pigments, generally yellow, red, and orange in color. Carotenoids include lycopene, β-carotene, and α-carotene, which are formed by cyclization of the two ends of lycopene. D’Ambrosio et al. (2004) produced tomato with a high β-carotene content in the fruit by overexpressing lycopene β-cyclase (*lcy-b*). The orange fruit had a stronger yellow color than normal tomato fruit. Lycopene is a red pigment that accumulates in fruit of tomato and other plant species, and is considered to play an important role in the removal of reactive oxygen species. In addition, lycopene has strong anticancer, photoprotective, antihypercholesterolemic, and antioxidant effects (Carvalho et al. 2021). Increase in the lycopene content is thought to be achieved by regulating the expression of crucial genes in the lycopene metabolic pathway. Although there are few examples of an increase in functional components achieved by loss of function, Luo et al. (2013) and Li X et al. (2018) succeeded in increasing the lycopene content by 4- to 5-fold. Luo et al. (2013) used RNA interference (RNAi) technology to suppress the expression of the tomato *STAY GREEN1* (*SlSGR1*) gene, which is involved in chlorophyll catabolism. The authors observed an increase in lycopene content as well as an effect on the ethylene synthesis pathway, which suppressed fruit ripening. Li X et al. (2018) used the CRISPR/Cas9 system to knockout several genes, including *SGR1*, to inhibit enzymes involved in the conversion of lycopene to β- and α-carotene. Consequently, the lycopene content was increased by approximately 5-fold and homozygous mutations were stably inherited in the subsequent generation.

#### a-3) GABA

Gamma-aminobutyric acid (GABA), a type of amino acid, is reported to have hypotensive and antistress effects. Blood pressure rises when sympathetic nerve activity increases. GABA is thought to suppress this sympathetic nervous system hyperactivity and reduce the secretion of noradrenaline, which works to constrict blood vessels, thereby lowering blood pressure (Nonaka et al. 2017). Therefore, GABA has attracted attention as a nutritional functional ingredient that contributes to the improvement of human health. The CRISPR/Cas9 system has been applied to generate mutations that resulted in an increase in GABA content (7- to 15-fold) in tomato fruit, but with a reduction in fruit number and yield (Nonaka et al. 2017). Sanatech Seed, a start-up company of the University of Tsukuba, began distribution of “Sicilian Rouge High Gaba”, a variety bred from a line developed using genome editing technology, as seedlings for cultivation in domestic vegetable gardens in October 2021.

### b) Rice

Astaxanthin, a red ketocarotenoid, is synthesized from β-carotene and exhibits exceptionally potent antioxidant properties. However, most higher plants lack the gene encoding β-carotene ketolase (*BKT*). Zhu et al. (2018) generated transgenic rice plants that accumulate astaxanthin in the endosperm by assembling and introducing four synthetic gene expression cassettes (maize *PSY1*, *Pantoea ananatis CrtI*, *Haematococcus pluvialis BHY*, and *Chlamydomonas reinhardtii BKT*) driven by a rice endosperm-specific promoter. A cross-section of the kernel shows that the pigments are uniformly distributed in the endosperm. In addition, the authors introduced a complete set of two maize regulatory genes (*ZmLc* and *ZmPl*) driven by endosperm-specific promoters and six structural genes of the anthocyanin biosynthesis pathway from *Coleus* (Lamiaceae) into purple rice to create transformant rice that accumulates anthocyanins in the endosperm (Zhu et al. 2017).

Deficiency of vitamin A, especially in children, remains a serious health problem in some developing countries. As a solution, genetically modified “golden rice” that accumulates large amounts of β-carotene, the precursor of vitamin A, in the grains was bioengineered. To achieve this, the genes phytoene synthase (*psy*) from daffodil (*Narcissus pseudonarcissus*) and phytoene desaturase (*crtI*) from the soil bacterium *Erwinia uredovora* were introduced into a rice cultivar (Peter et al. 2002). The accumulation of β-carotene was further dramatically increased by alternatively using the *psy* gene from maize (Paine et al. 2005). In addition, an intensive breeding program has successfully produced locally adapted varieties in Asia (Swamy et al. 2021). Australia and New Zealand Food Standards, Health Canada, US Food and Drug Administration, and the Philippine Department of Agriculture-Bureau of Plant Industry have completed risk assessments and, in July 2021, the Philippines became the first country to approve “golden rice” for commercial production.

### c) Oilseed crops

Polyunsaturated fatty acids (PUFA) include ω-3 fatty acids and ω-6 fatty acids. Alpha-linolenic acid (ω-3 type) and linoleic acid (ω-6 type) are essential fatty acids for humans. Long-chain PUFAs, such as eicosapentaenoic acid (EPA) and docosahexaenoic acid (DHA), can be synthesized in the human body but the quantities are insufficient to meet requirements. Daily intake of EPA and DHA reduces the risk of heart disease, Alzheimer’s disease, bipolar disorder, schizophrenia, type 2 diabetes, and other disorders (Patel et al. 2021). In addition, DHA is essential for proper visual and neurological development after birth (Lauritzen et al. 2016). Fish oil and seafood are sources of ω fatty acids, but owing to issues such as resource depletion, development of alternative sources is desirable. Plants, especially seed crops, are good sources of trace amounts of PUFAs, but these lack desaturase and elongase, which are necessary for EPA and DHA synthesis, and thus cannot synthesize these long-chain fatty acids. Genetic modification of seed crops for high oleic acid content has been achieved in sunflower, oilseed rape, soybean, and flaxseed, which warrant attention for long-chain PUFA synthesis.

The GM canola lines LBFLFK and NS-B50027-4 harboring multiple desaturase and elongase genes have been developed by BASF and Cargill, and Nuseed and the Commonwealth Scientific and Industrial Research Organisation (CSIRO), respectively (Napier et al. 2019; Petrie et al. 2020). Comparing these two lines, LBFLFK accumulates higher quantities of EPA and NS-B50027-4 accumulates greater amounts of DHA. Specifically, LBFLFK produces seed oil containing ∼7% EPA, ∼3% DPA, and ∼1% DHA, whereas NS-B50027-4 produces oil containing <0.5% EPA, ∼1% DPA, and ∼10% DHA.

Rothamsted Research in the UK has engineered the oilseed crop *Camelina sativa* by introducing five and seven foreign genes from microalgae to increase EPA and DHA contents, respectively (Ruiz-Lopez et al. 2014). These genes are under the control of seed-specific promoters, ensuring that fatty acids are synthesized during seed development. These transgenic lines demonstrate significant increases in EPA and DHA contents, and thus are a viable and sustainable source of ω-3 long-chain PUFAs comparable to the quantities in fish oil.

## Improvement of photosynthesis

The rate of increase in crop yields achieved during the twentieth century has slowed and traditional plant breeding is reaching a plateau. To meet the increasing demand for food from a growing global population, it is crucial to use innovative tools and biotechnological solutions to improve the efficiency of light-to-biomass conversion in plants (Batista-Silva et al. 2020; Ort et al. 2015; Smith et al. 2023). Improving photosynthetic capacity is crucial to increase crop yields. Synthetic biology offers an ideal approach to enhance complex metabolic systems, which are a bottleneck for photosynthesis. Several review articles have suggested synthetic biology approaches to enhance photosynthetic capacity (da Fonseca-Pereira et al. 2022; Kubis and Bar-Even 2019; Zhu et al. 2020). Here, we summarize some of the leading synthetic biology challenges to date to improve photosynthesis bottlenecks in plants and cyanobacteria.

### a) Rubisco engineering

Rubisco, a critical enzyme in the Calvin–Benson–Bassham (CBB) cycle, catalyzes the carboxylation of ribulose-1,5-bisphosphate (RuBP) with CO_2_ during the RuBP carboxylase reaction to produce two molecules of 3-phosphoglycerate (3PGA). However, the inherent inability of Rubisco to efficiently discriminate oxygen and CO_2_ leads to energy waste during photorespiration. Plants have evolved a slower Rubisco variant with improved CO_2_/O_2_ discrimination to adapt to the increase in atmospheric O_2_ concentration (Gionfriddo et al. 2024).

The rate of photosynthesis in C3 plants under current atmospheric conditions is limited by Rubisco due to its extremely low catalytic rate and low affinity for CO_2_. In recent years, the concentration of atmospheric carbon dioxide has continued to increase at a faster rate than plants can adapt. The replacement of C3 plant Rubisco with a variant that has a faster catalytic rate is a potential strategy to enhance carbon fixation (Orr et al. 2016). Lin et al. (2014) successfully replaced tobacco Rubisco with a faster cyanobacterial enzyme via chloroplast transformation. The tobacco chloroplast *rbcL* gene encoding the Rubisco large subunit was replaced by inserting the large and small subunit (*rbcLS*) genes from the cyanobacterium *Synechococcus elongatus* PCC 7942. Although cyanobacterial Rubisco assembles correctly in tobacco chloroplasts, the resulting transplastomic plants grew only under high CO_2_ concentrations. Tobacco and potato plants expressing Rubisco from photosynthetic bacteria and red algae, respectively, grew only under an elevated CO_2_ atmosphere (Gunn et al. 2020; Manning et al. 2023; Wilson et al. 2016). These results indicate that the concomitant introduction of CO_2_-concentrating mechanisms (CCMs) into the C3 plant chloroplast is essential for the benefit of Rubisco replacement to be obtained. Recently, engineering efforts have targeted the small subunits of Rubisco to enhance catalytic rates in tobacco, rice, and Arabidopsis (Mao et al. 2023). Matsumura et al. (2020) overexpressed a RbcS from sorghum (a C_4_ plant) in rice, in which the production of endogenous RbcS was disrupted by CRISPR/Cas9 technology. The transgenic rice accumulated Rubisco composed of rice RbcL and sorghum RbcS, but the level was reduced by up to 67% compared to the wild-type rice. The hybrid Rubisco exhibited a lower affinity for CO_2_ but a faster catalytic rate in vitro under high CO_2_ concentrations, as in C4 plant Rubisco. However, the transgenic rice showed a lower leaf photosynthesis rate under ambient CO_2_ conditions than the wild-type plants because of less Rubisco content and enzyme properties.

### b) Introduction of photorespiratory bypass into chloroplasts

Under current conditions for C_3_ plants growing at 25°C, approximately 25% of Rubisco reactions use O_2_ instead of CO_2_, resulting in the oxygenation of RuBP to produce one molecule each of 3PGA and 2-phosphoglycolate (2PG). However, the two carbon atoms of 2PG cannot be metabolized in the CBB cycle. The recycle of 2PG to 3PGA takes place through the photorespiratory pathway (Bauwe 2023). During photorespiration, one of the four carbon atoms in two molecules of 2PG molecules is released as CO_2_, accompanied by the release of ammonia and the consumption of ATP and NAD(P)H. Photorespiration significantly reduces the efficiency of carbon assimilation in C_3_ plants, resulting in yield losses of approximately 30% or more (Smith et al. 2023). To improve 2PG recovery and photosynthetic efficiency, several photorespiratory bypasses have been introduced into plant chloroplasts (Kebeish et al. 2007; Maier et al. 2012; South et al. 2019).

To evaluate the efficacy of alternative photorespiratory pathways in improving the productivity of C_3_ field crops, South et al. (2019) performed a comparative analysis of three photorespiratory bypass pathways (AP1–AP3) in tobacco. AP1 utilized five genes from the *E. coli* glycolate oxidation pathway, AP2 followed the pathway of Maier et al. (2012) using plant glycolate oxidase and malate synthase and *E. coli* catalase, and AP3 employed plant malate synthase and a green algal glycolate dehydrogenase. In the alternative pathways, glycolate metabolism and photorespiratory CO_2_ release occur in the chloroplast. Theoretically, the CO_2_ release results in an increase in the CO_2_ partial pressure in the chloroplast, enhancing photosynthesis. Furthermore, the consumption of ATP for the recovery of ammonia is predicted to be decreased by the suppression of the flux to the native pathway. The enzymes in these designs were targeted to tobacco chloroplasts. In addition, RNAi technology was used to downregulate a native chloroplast glycolate transporter in the photorespiratory pathway to limit metabolite flux through the native pathway. Field trials revealed a significant 20–24% increase in biomass of AP3-introduced tobacco plants with RNAi compared with that of the wild type, accompanied by a 17% improvement in light-use efficiency of photosynthesis in the field.

### c) Introduction of CCM into plants

In cyanobacterial CCMs, Rubisco is spatially close to carbonic anhydrase, increasing the cellular bicarbonate concentration via transporters. Encapsulation of Rubisco and carbonic anhydrase in carboxysomes allows efficient CO_2_ supply for RuBP carboxylation. Heterologous CCM construction in plant chloroplasts, as proposed by Rottet et al. (2021), has the potential to enhance photosynthesis in C_3_ plants. Recent progress includes the generation of Rubisco-containing carboxysomes in tobacco chloroplasts (Chen et al. 2023; Long et al. 2018). However, replacement of the endogenous tobacco *rbcL* gene with the genes for Rubisco and key carboxysome structural proteins from the cyanobacterium *Cyanobium marinum* PCC 7001 by chloroplast transformation resulted in low growth rates among the transgenic plants, even when grown under 2% CO_2_. This result indicates that functional encapsulation of Rubisco in the carboxysome is possible in the chloroplast. However, the simultaneous encapsulation of carbonic anhydrase as well into the carboxysome still remains a significant challenge. Rolland et al. (2016) successfully targeted cyanobacterial bicarbonate transporters to the chloroplast envelope in *Nicotiana benthamiana* cells. The challenge ahead is to integrate these components as a functional chloroplastic CCM.

### d) Introduction of the C_4_ pathway to C_3_ plants

Similar to the CCM of cyanobacteria, C_4_ plants have evolved C_4_ metabolism to reduce the oxygenase activity of Rubisco. C_4_ photosynthesis involves coordinated metabolism in mesophyll cells (MCs) and bundle sheath cells (BSCs). Phosphoenolpyruvate carboxylase initiates CO_2_ assimilation in MCs, and the resulting C_4_ acids are transported and decarboxylated in BSCs, increasing the CO_2_ partial pressure around Rubisco (Furbank 2011). Given its superior nitrogen- and water-use efficiency, the transfer of C_4_ traits to enhance C_3_ photosynthesis has long been desired (Ermakova et al. 2020; Furbank et al. 2023). However, substantial changes to the chloroplast proteome in MCs and BSCs are required to implement full C_4_ biochemistry in a C_3_ plant, thus presenting challenges for this strategy. Ermakova et al. (2020) proposed the introduction of a minimal C_4_ cycle in rice by incorporating maize enzymes into specific leaf cells. This minimal cycle aims to increase the CO_2_ partial pressure around Rubisco in BSCs, potentially benefiting plants with C_3_ leaf anatomy. The transformation vector included coding regions for maize carbonic anhydrase, phosphoenolpyruvate carboxylase, NADP-malate dehydrogenase, pyruvate orthophosphate dikinase, and NADP-malic enzyme, all under the control of cell-preferential promoters (Ermakova et al. 2021). The resultant transgenic plants exhibited the low malate-to-phosphoenolpyruvate flux associated with C4 carboxylation, as confirmed by ^13^CO_2_ labeling. However, no effect on photosynthetic rate was observed. It has been proposed that C4 plants have acquired the complex traits required for C4 photosynthesis through a multistep evolutionary process. This process has involved the repositioning of mitochondria in BS cells, the development of the Kranz anatomy, the evolution of a two-celled photorespiratory concentration mechanism, and the development of a high vein density (Sage et al. 2012), suggesting that significant challenges remain in the artificial conversion of C3 photosynthesis to C4 photosynthesis due to its inherent complexity.

### e) Design of synthetic carbon fixation pathway

Carbon fixation is essential for the conversion of CO_2_ into biomass and involves a complex enzymatic pathway. During billions of years of evolution in plants, algae, and microorganisms, the efficiency of carboxylating enzymes is a known bottleneck in natural CO_2_ fixation. Schwander et al. (2016) identified enoyl-CoA carboxylases/reductases as highly efficient CO_2_-fixing enzymes that are not naturally used in the evolutionary processes of autotrophs. Based on this finding, these authors constructed an optimal synthetic carbon fixation pathway in vitro, known as the crotonyl-CoA/ethylmalonyl-CoA/hydroxybutyryl-CoA (CETCH) cycle, using 17 enzymes (including three artificial enzymes) isolated from different organisms. This pathway, validated by a DBTL cycle in synthetic biology, showed up to five-times higher efficiency than the in vivo rate for the common natural carbon fixation pathway. The concept of metabolic proofreading is rarely considered in synthetic pathway design and indicates that this design principle should be more systematically incorporated into synthetic biology. The successful in vitro reconstitution of a synthetic enzyme network for CO_2_ conversion to organic products, which outperforms chemical processes, lays a foundation for several future applications. Miller et al. (2020) used microfluidics to develop chloroplast-mimicking devices that encapsulate and function as photosynthetic membranes in cell-sized droplets (CETECH cycle ver. 6.0). Similar methods have been used to further optimize these and other metabolic pathways for many biotechnological applications.

### f) Regulation of photosynthetic light reactions

The photosynthetically active band, ranging from 400 to 740 nm, accounts for 48.7% of the total solar energy received at the Earth’s surface. Thus, higher plants cannot use 51.3% of the solar energy. Furthermore, approximately 10% of this radiation is reflected, resulting in a minimum loss of 4.9%. Approximately 6.6% of the incident solar energy is irreversibly lost as heat owing to relaxation of higher excited states of chlorophyll (Zhu et al. 2008). As a result, only 40% of the solar energy is used for photosynthesis (Leister 2019). The theoretical maximum efficiency of light reactions is 26%. Given energy losses in carbohydrate biosynthesis and photorespiration, the theoretical maximum photosynthetic energy conversion efficiency is 4.6% for C_3_ plants and 6% for C_4_ plants. Nevertheless, field crops typically achieve efficiencies of less than 1%, suggesting there is substantial potential to enhance the photosynthetic light response (Zhu et al. 2008).

Plants dissipate excess sunlight as heat for protection, a process termed thermal dissipation. This is monitored as the energy-dependent quenching component of nonphotochemical quenching (NPQ) of chlorophyll fluorescence (Müller et al. 2001). Thermal dissipation reduces the photosynthetic efficiency for a period of minutes, especially when the leaves are shaded. The regulatory mechanism for photoprotection during light transitions is a potential target to enhance photosynthesis. Under high light intensity, NPQ levels are positively correlated with the abundance of PsbS protein and further stimulated by de-epoxidation of violaxanthin to antheraxanthin and zeaxanthin catalyzed by violaxanthin de-epoxidase (VDE). Upon the transition to low light intensity, the rate of NPQ relaxation correlates with the zeaxanthin epoxidation rate, which is catalyzed by zeaxanthin epoxidase (ZEP) (Müller et al. 2001). Transformation of tobacco plants with the Arabidopsis *VDE*, *ZEP*, and *PsbS* coding sequences under the control of different promoters for expression in leaves resulted in faster NPQ relaxation, an increase in CO_2_ fixation rate, and 14–20% higher biomass productivity under field conditions compared with the control plants (Kromdijk et al. 2016). Optimizing the transition between light energy utilization and photoprotection is expected to increase overall light-use efficiency by redirecting energy to photochemistry (Slattery and Ort 2021).

Several attempts have been reported to regulate photosynthetic electron transport by introducing soluble electron transporters that have been lost in host plants during evolution. Expression of cyanobacterial flavodoxin, an electron carrier downstream of photosystem I (PSI), partially replaced the plant ferredoxin function and conferred broad stress tolerance to tobacco (Blanco et al. 2011; Tognetti et al. 2006, 2007). Similarly, expression of red algal cytochrome *c*_6_, an electron carrier heme protein from the cytochrome *b*_6_*f* complex to PSI, enhanced Arabidopsis growth and photosynthesis (Chida et al. 2007). Flavodiiron proteins, which are conserved from cyanobacteria to gymnosperms, reduce dioxygen directly to water using ferredoxin and/or NAD(P)H as electron donors and act as a safety valve for electrons downstream of PSI. To dissipate excess reducing power from the photosynthetic electron transport chain, flavodiiron proteins from the moss *Physcomitrella patens* were introduced to Arabidopsis and rice (Wada et al. 2018; Yamamoto et al. 2016). Overexpression of flavodiiron proteins protects PSI from photodamage under fluctuating light.

## Lignocellulose engineering

There is an urgent need to transition from a global economy based on fossil resources, which results in greenhouse gas emissions, to one based on biorefineries, which use lignocellulosic biomass to produce fuels, chemicals, and materials. Wood serves as a crucial reservoir of lignocellulosic biomass, consisting primarily of secondary thickened cell walls rich in cellulose, hemicellulose, and lignin. Cellulose is a raw material for the pulp and paper industry, and cellulose and hemicellulose can be decomposed into monosaccharides and fermented to produce bioethanol, lactic acid, and detergents. Given that lignin adversely affects the efficiency of wood processing for these applications, trees can be engineered to less lignin for the production of paper and fermentable sugars. Antisense or RNAi can reduce the activity of any step in the lignin biosynthetic pathway from phenylalanine ammonia-lyase (PAL) to cinnamyl alcohol dehydrogenase (Chanoca et al. 2019). Several variables influence the reduction in lignin content, including the degree of downregulation of target genes and enzyme activities, which depend on the efficiency of the silencing construct used, the size of the gene family, and the redundancy within the gene family. In general, downregulation at the cinnamate 4-hydroxylase (C4H) to cinnamoyl-CoA reductase stage can reduce lignin contents more dramatically. One means of lignin engineering in trees, enabled by CRISPR/Cas9 editing, is to edit multiple genes simultaneously (allele stacking) to optimize biomass processing efficiency. In Arabidopsis, stacking three double mutations (*tra* *comt*, *c4h comt*, and *4cl comt*) led to improved saccharification efficiency owing to the reduced lignin content and increased fractions of guaiacyl and 5-hydroxyguaiacyl guaiacyl (de Vries et al. 2018). Quantitative and integrated multiomics analysis of lignin biosynthesis can also advance the strategic engineering of wood for wood itself, pulp, and biofuels. At least 21 genes in poplar participate in the wood-forming pathway, encoding enzymes that mediate 37 reactions for 24 metabolites, leading to lignin biosynthesis and affecting wood properties. Wang JP et al. (2018) perturbed these 21 pathway genes and integrated transcriptomic, proteomic, fluxomic, and phenomic data from 221 lines selected from approximately 2,000 transgenic plants. From this analysis, the authors estimated how changes in expression of pathway genes or gene combinations affected 25 tree traits, including protein abundance, metabolic flow, metabolite concentration, tree growth, density, strength, and saccharification. The authors also analyzed the effects of simultaneous down-regulation of PALs and caffeoyl-CoA 3-*O*-methyltransferases (CCoAOMTs) or that of PAL, C3′H, and CCoAOMT on improvement of wood properties and sugar release (Wang JP et al. 2018). A more comprehensive and detailed review on this topic is presented in a separate article in this issue (Yoshida et al. 2024).

## Modification of secondary metabolites in plants

Manipulation of secondary metabolism in plants has been performed to understand secondary metabolite biosynthesis and its regulatory mechanisms, produce useful metabolites in medicinal plants, decrease antinutritional metabolites in crops, and modify the appearance of houseplants, for example. Flower coloration is among the most important traits for flower breeding. Many studies have reported the generation of flowering plants with altered coloration by disruption of pigment biosynthetic genes (Nitarska et al. 2021; Tasaki et al. 2019; Yu et al. 2021). Alteration of pigment production is also useful for fundamental research, including as a visible marker for transformation (He et al. 2020) and visualization of root colonization by arbuscular mycorrhizal fungi (Kumar et al. 2022). Similar strategies have been applied to modify fragrance in plants. Mutation of *BETAINE ALDEHYDE DEHYDROGENASE* (*OsBADH2*) induced by CRISPR/Cas9 editing changed a non-aromatic rice variety to an aromatic variety (Ashokkumar et al. 2020). Overexpression of heterologous terpene synthesis genes increased and altered the fragrance in tobacco (Lücker et al. 2004).

Research on secondary metabolite production in cultured tissues started more than 60 years ago and has evolved with the development of new biotechnologies, such as transformation, omics analysis, and genome editing (Misawa 1994; Ozyigit et al. 2023; Saito and Matsuda 2010). One bottleneck for plant secondary metabolite production is tissue- and environment-dependency of their biosynthesis. Optimization of culture systems and modification of metabolic pathways assists in overcoming this obstacle. *Lithospermum erythrorhizon* produces shikonin, a naphthoquinone pigment with various bioactivities (Yadav et al. 2022). This compound was among the first to be produced by tissue culture on an industrial scale (Tabata and Fujita 1985). *Lithospermum erythrorhizon* cultured cells produce shikonin in M9 medium in the dark, whereas Murashige and Skoog medium (rich in NH_4_^+^ and low in Cu^2+^) and light negatively regulate shikonin production (Yazaki 2017). Another example is a natural sweetener, glycyrrhizin, isolated from licorice (*Glycyrrhiza* spp.). Glycyrrhizin is accumulated in the thickened roots of licorice plants grown for 2–3 years. Although cultured tissues, suspension-cultured cells, liquid-cultured stolons, and hairy roots have been established, they accumulate no or only trace amounts of glycyrrhizin (Hayashi et al. 1988; Kojoma et al. 2010; Mousa et al. 2007; Tamura et al. 2018). Chiyo et al. (2024) successfully established glycyrrhizin-producing hairy root lines by simultaneously overexpressing *CYP88D6*, the bottleneck in glycyrrhizin biosynthesis, and knocking-out competitive soyasaponin biosynthetic pathways.

## Transfer of secondary metabolite pathways into microorganisms

Fermentation, traditionally used in food and beverage production, has evolved into an industrial process for manufacturing various chemicals, pharmaceuticals, enzymes, proteins, and biofuels using microorganisms. Precision fermentation, a burgeoning field that is gaining traction in food technology, employs microorganisms as “cell factories” to produce specific functional ingredients. Start-ups in the USA, such as Perfect Day, ClaraFoods, and Impossible Foods, are utilizing precision fermentation to produce proteins and flavor-enhancing meat substitutes. By reconstituting plant-specific metabolic pathways in microorganisms, alternative production of the plant metabolites has been achieved. Examples include terpenoids, flavonoids, and alkaloids produced using yeast or *E. coli* as hosts (Birchfield and McIntosh 2020; Pyne et al. 2019). This technology shows promise for sustainable, large-scale production of diverse compounds.

### a) Terpenoids

Terpenoids, derived from isoprene units, form a diverse class of compounds crucial for plant physiology and contribute to the aroma, taste, and color. With distinct skeletons and functional groups, terpenoids are utilized in pharmaceuticals, biofuels, flavors, and fragrances. Microbial cell factories, employing organisms such as *E. coli* and yeast, offer sustainable and efficient production of high-value terpenoids. Genetic tools, such as metabolic engineering and genome editing, show promise for generating novel terpenes. In 2013, the antimalarial drug artemisinin attracted attention when its precursor was biosynthesized in yeast using a synthetic biology approach (Paddon et al. 2013). Artemisinin is extracted from the asteraceous plant *Artemisia annua* and is highly effective against the malaria parasite. Paddon et al. (2013) achieved production of 25 g l^−1^ of artemisinic acid from transgenic yeast by overexpressing multiple genes in the farnesyl pyrophosphate pathway, introducing six genes in the artemisinic acid synthesis pathway, and suppressing ERG9, the branching point of artemisinic acid and sterol biosynthesis. The yeast-produced artemisinic acid was chemically converted to artemisinin, which is more cost-effective and environmentally friendly than total synthesis. In addition to artemisinin, many other terpenoids important for plant-derived fragrances have been produced in yeast and *E. coli* using synthetic biology-based methods. Geraniol, limonene, and farnesene neronidol have been produced in yeast, while pinene, limonene, bisabolol, and longifolene have been produced in *E. coli* (Zhang and Hong 2020).

Terpene glycosides could be served as alternatives to sugar for human dietary needs. Xu et al. (2022) outlined the benefits and hurdles of precision fermentation for natural sugar substitutes, categorizing them into five types based on their molecular structure. These include monosaccharides such as *N*-acetylglucosamine, *D*-allulose, and *D*-tagatose; oligosaccharides such as 2′-fucosyllactose and trehalose; sugar alcohols such as erythritol and xylitol; and terpene glycosides such as steviol glycosides and glycyrrhizin. Traditional extraction methods are costly and environmentally unfriendly because of the low natural sugar substitutes content and climate dependency. Microbial cell factories offer a promising solution, especially for terpene glycosides, given their high productivity, environmental sustainability, and controllability, making them a potential future standard (Qu et al. 2023).

The main steviol glycosides in *Stevia rebaudiana* are stevioside and rebaudioside A (RebA), which are >250 times sweeter than sucrose but have a lingering bitter aftertaste (DuBois and Stephenson 1985; Prakash et al. 2014). Production of the minor compounds RebD and RebM is desirable because they are sweeter but less bitter than stevioside and RebA. In stevia plants, UGT76G1 catalyzes the conversion of RebD to RebM. However, UGT76G1 also produces RebG, RebQ, RebI, and 1,3-bioside, which cannot be converted to RebD and RebM (Olsson et al. 2016). These authors predicted the 38 residues in the catalytic domains potentially involved in product selectivity by molecular-docking simulation. As a result of functional screening of the mutagenized UGT76G1 at these residues, UGT76G1 was engineered to achieve selective production of RebD and RebM in yeast. The yeast-produced next-generation sweetener EverSweet™ was commercialized in the USA in 2019.

In addition to its use as a medicine, licorice is widely used as a sweetening ingredient in food products, such as seasonings and confectionery. Glycyrrhetinic acid (GA), an aglycon of glycyrrhizin, a sweetener produced in licorice roots, has excellent anti-inflammatory activity, and is widely used in the pharmaceutical and cosmetic industries. Current industrial production involves acid hydrolysis of glycyrrhizin extracted from wild licorice plants (Mukhopadhyay and Panja 2008), which is environmentally hazardous and destroys agricultural land. Wang et al. (2019) innovatively engineered yeast strains by introducing optimized genes encoding β-amyrin 11-oxidase, 11-oxo-beta-amyrin 30-oxidase, β-amylin synthase, and Arabidopsis NADPH cytochrome P450 reductase, to achieve production of 2.5 mg l^−1^ of β-amylin and 14 µg l^−1^ of GA. Furthermore, the addition of licorice-derived cytochrome b5 (GuCYB5) enhanced GA production 8-fold. Combining 10 yeast mevalonate pathway genes with GuCYB5 further increased GA production 40-fold during batch fermentation (Wang et al. 2019). Chung et al. (2020) and Jozwiak et al. (2020) identified the last piece of glycyrrhizin biosynthesis, namely, cellulose synthase-derived glycosyltransferase (CSyGT) attaching the first glucuronide molecule to the C-3 position of GA and other triterpene aglycons. These authors achieved de novo production of glycyrrhizin in yeast and tobacco platforms. Glycyrrhetinic acid-3-*O*-monoglucronide (GAM) shows stronger sweetness than glycyrrhizin (Yang et al. 2019). Although GAM is a minor compound in licorice plants, its selective production was achieved in the yeast strain expressing glycyrrhizin biosynthetic enzymes other than GuUGT73P12, which converts GAM to glycyrrhizin in planta (Chung et al. 2020).

### b) Alkaloids

Alkaloid is nitrogen-containing secondary metabolites. Many alkaloids are toxic to other organisms and often exhibit pharmacological effects, and thus are used as drugs or medicines. Alkaloid-derived drugs are generally semi-synthesized from starting materials extracted from plants, resulting in limited supply and high market prices. Recent advances in synthetic biology have made it possible to transfer the biosynthetic pathway consisting of several enzyme genes into microorganisms.

Tropane alkaloids are produced by plants of the genera *Atropa*, *Duboisia*, and other members of the Solanaceae, and are used to treat a variety of neurological disorders owing to their anticholinergic properties. Tropin, a crucial intermediate in the biosynthetic pathway of scopolamine and other medicinal tropane alkaloids, was successfully produced in a novel manner from simple carbon and nitrogen sources in yeast (Srinivasan and Smolke 2019). The engineered strain produced tropin at a titer of 6 mg l^−1^ with the addition of 15 genes, including 11 genes from diverse plants and bacteria, and seven disruptions of yeast regulatory and biosynthetic genes.

Opioids, extracted from opium poppy, are important medicines, including the painkiller morphine and the cough suppressant codeine. (*S*)-Reticuline is the important intermediate of many isoquinoline alkaloids such as berberine, morphine and codeine. Minami et al. (2008) achieved the production of 55 mg l^−1^ of (*S*)-reticuline by incubating dopamine with crude enzymes expressed in *E. coli*. Subsequently, they demonstrated the synthesis of various (*S*)-reticuline-derived alkaloids through combination culture of transgenic *E. coli* and yeast. Nakagawa et al. (2011) successfully engineered *E. coli* strains capable of producing 46 mg l^−1^ of (*S*)-reticuline using a simple carbon source, without the need for costly substrate additives. The opioid compounds thebaine and hydrocodone have been biosynthesized from monosaccharides in yeast (Galanie et al. 2015). Thebaine (6.4 µg l^−1^) was obtained by expressing 21 genes introduced from plants, mammals, and bacteria, overexpressing two types of yeast genes, and inactivating one type of yeast gene. Two additional bacterial genes were introduced into thebaine biosynthetic bacteria, resulting in hydrocodone biosynthesis but at a low concentration (approximately 0.3 µg l^−1^) (Galanie et al. 2015). Noscapine, a promising anticancer drug, synthesized from canadine was recently shown to be encoded by a cluster of 10 genes from opium poppy. More than 30 enzymatic genes from plants, bacteria, mammals, and yeast, including seven plant enzymes localized to the endoplasmic reticulum, were reconstructed and successfully introduced into yeast (Li Y et al. 2018). By adjusting and optimizing the expression levels of the enzymes, the host’s endogenous metabolic pathway, and fermentation conditions, the titer of noscapine was increased to 2.2 mg l^−1^, more than 18,000 times higher than the initial noscapine titer. Furthermore, microbial production of halogenated benzylisoquinoline alkaloids was demonstrated by feeding the optimized noscapine-producing strain with modified tyrosine derivatives (Li Y et al. 2018).

### c) Cannabinoids

Cannabinoids derived from cannabis, primarily tetrahydrocannabinol and cannabidiol, are not clinically used as anticancer agents, but can reduce pain from cancer and chemotherapy. These cannabinoids have been approved as antiemetics, analgesics, pain relievers, and antispasmodics. In yeast, the major cannabinoids cannabigerolic acid, Δ9-tetrahydrocannabinolic acid, cannabidiolic acid, Δ9-tetrahydrocannabivarinolic acid, and cannabidivarinic acid were completely biosynthesized from galactose (Luo et al. 2019). In this study, the mevalonic acid pathway was modified to supply high concentrations of geranyl pyrophosphate, and a heterologous multispecies hexanoyl-CoA biosynthetic pathway was introduced. A cannabis gene encoding an enzyme involved in the biosynthesis of olivetolic acid, as well as a gene for an undiscovered enzyme with geranyl pyrophosphate:olivetolic acid geranyltransferase activity and a corresponding gene for a cannabinoid synthase were introduced. Furthermore, a biosynthetic approach to produce cannabinoid analogues was established by using a combination of several pathway genes.

### d) Phenolic compounds

Plants produce diverse phenolic compounds including lignin and flavonoids. Flavonoids are a group of phenolic compounds synthesized through the phenylpropanoid and polyketide pathways. Typical examples include tea catechins, soy isoflavones, and anthocyanins responsible for flower colors. Some flavonoids exhibit antioxidant and hormone-like effects and are attracting attention as functionally active ingredients to maintain and promote human health.

Anthocyanin synthesis in microorganisms has been achieved by simultaneously culturing four *E. coli* strains in which 15 different enzymes and transcription factors from plants and other microorganisms are separately expressed (Jones et al. 2017). The first strain produced precursors of phenylpropanoic acid used in chalcone synthesis from xylose, glucose, and glycerol; the second strain produced flavanones; the third strain converted these to dihydroflavonol; and the fourth strain produced 9.5 mg l^−1^ of the anthocyanin calistophine (pelargonidin). This is a landmark study that provides the basis for a major advance in strain and process design.

Breviscapine, the total flavonoid extract of *Erigeron breviscapus* is widely used in the treatment of cerebral infarction and its sequelae, cerebral thrombosis, coronary heart disease, and angina pectoris. Two critical enzymes in the biosynthetic pathway (flavonoid 7-*O*-glucuronosyltransferase and flavone-6-hydroxylase) were identified from *E. breviscapus* genome and the production of breviscapine from glucose using yeast was achieved (Liu et al. 2018). Using metabolic engineering and sulfidic culture, the active components of breviscapine, scutellarin and apigenin-7-*O*-glucoside, were obtained at concentrations of 108 mg l^−1^ and 185 mg l^−1^, respectively.

Artepillin C, a prenylated phenylpropane, shows a broad spectrum of pharmacological properties. Munakata et al. (2019) identified *Artemisia capillaris* prenyltransferase 1 (AcPT1) enzyme which catalyzes the stepwise transfer of two prenyl residues to *p*-coumaric acid, leading to the synthesis of drupanin (monoprenyl) and artepillin C (diprenyl). They engineered yeast strains capable of synthesizing artepillin C de novo from *p*-coumaric acid and DMAPP, achieving a yield of 6.8±0.6 µmol l^−1^. However, 99% of *p*-coumaric acid existed in the medium but not in the cells, suggesting that *p*-coumaric acid was not effectively converted into duparin and artepillin C and was released in the medium. Then, they supplemented the medium with an excess amount of *p*-coumaric acid, resulting in a higher total production of drupanin and artepillin C (113±7 µmol l^−1^).

## Biosensors

Biosensors (genetically encoded sensors) are the first elements of the genetic circuit and a transformative field of synthetic biology with potential applications in agriculture (Goold et al. 2018). A biosensor is a genetically encoded element, such as a promoter or protein, that provides an output and causes gene expression in response to an exogenous stimulus. For example, microbial biosensors detect organic acids, carbohydrates, coenzyme B12, heavy metals, amino acids, light, pathogens, and plant hormones (Goold et al. 2018). In plants, examples of chemical-stimuli-driven stress-resistant plants are reported. Transgenic Arabidopsis expressing the fungicide mannidipropamide-sensitive PRY1 receptor exhibits an abscisic acid-like response to resist water deficiency following spray application of the fungicide, causing stomatal opening and other defense responses (Park et al. 2015). Other types of receptors have likewise been reconstituted in plants to generate synthetic switches. For example, transcriptionally regulated synthetic sensors that monitor cytokinin signaling in plants by expression of fluorescent proteins (Zürcher et al. 2013) and light-sensitive gene switches that turn gene expression on or off in response to red or far-red light have been reported (Müller et al. 2014).

Plant sentinels (originally, “sentinel” plants) include susceptible plants that exhibit visible symptoms of infection more rapidly. They are introduced into at-risk populations and serve as early warning beacons of infection and may be incorporated into surveillance programs. However, while sentinel hosts can detect pathogens earlier because of the faster progression of disease, there is a negative aspect in that faster disease progression promotes earlier transmission (Lovell-Read et al. 2023). Another example of plant sentinels is the biosensor plants that have been modified to detect the presence of specific components in their immediate environment and send signals. For example, by encoding synthetic signaling pathways with modular receptors, plants can be engineered to respond to a wide variety of environmental factors, such as environmental pollutants, nutrients, and abiotic stresses (Goold et al. 2018). A plant biosensor for the explosive 2,4,6-trinitrotorune (TNT) was created with bacterial-derived receptors, transmembrane kinases, and response regulators for TNT (Antunes et al. 2011). When the plant is exposed to TNT, a transmembrane signal activator activates a synthetic promoter, which transcribes a gene that inhibits chlorophyll synthesis and initiates chlorophyll degradation. This synthetic signaling system is among the first successful examples generated using modular assembly of bacterial and plant protein domains, and refinement of the signaling components allows for more powerful and specific signaling pathways (Antunes et al. 2011). Other sentry plants have been developed that detect exposure to gamma rays (Peng et al. 2014). Another example is Arabidopsis in which the promoter region of a gene (*SQD1*) whose expression increases in response to phosphorus deficiency is linked to a reporter gene (Hammond et al. 2003). Similar improvements can be applied to field crops to precisely and visually direct the temporal and spatial feeding of fertilizers, improve application methods, reduce waste, and increase sustainability.

## Nitrogen fixation

Plants depend on beneficial interactions between roots and root-associated microorganisms, which form symbiotic relationships with certain bacteria and filamentous fungi to obtain essential plant nutrients, such as nitrogen and phosphorus. Some symbiotic microorganisms also enhance the immune response of plants, conferring the ability to the plants to control pathogens. These microorganisms can be isolated, genetically engineered to have desirable traits, reconstituted as a synthetic microbial community, and then inoculated with the genetically engineered strain on the plant, which can then recolonize the host.

Using this method, Bloch et al. (2020) characterized strains of plant-associated proteobacteria from maize roots with regard to their nitrogen regulatory networks. To optimize nitrogen fixation in maize, crucial genes in the synthesis pathway of this microbe were subjected to genome editing and modified to fix and release nitrogen in the presence of exogenous nitrogen sources. While the wild-type strain suppressed nitrogen fixation in the presence of abundant bioavailable nitrogen, such as in greenhouse and field fertilization experiments, the modified strain showed increased expression of nitrogenase in the rhizosphere of greenhouse and field maize, even in the presence of exogenous nitrogen. Such strains may be outside the regulatory framework for genetic modification and could be used for commercial applications to supply fixed nitrogen to grain crops (Bloch et al. 2020).

More directly, to perform nitrogen fixation in the plant itself, Allen et al. (2017) introduced into tobacco 16 genes with mitochondria-targeting signal for proteins comprising nitrogenases from diazotrophs. These proteins were correctly translocated into the mitochondrial matrix but actual enzymatic activities were not reported. Because mitochondoria contain enzymes consuming oxygen, the expression of oxygen-hypersensitive enzyme such as nitrogenase in mitochondria would be a good strategy and actually a possibility to reconstitute complete components of nitrogenases in plants was demonstrated (Allen et al. 2017).

## Luminescent plants

Bioluminescence is known in bacteria, fungi, and animals, such as *Photobacterium leiognathi*, *Neonothopanus nambi*, firefly, various fishes, and jellyfish. Luciferase and luciferin have been widely utilized for in vitro reporter (Sherf et al. 1996) and in vivo imaging (Evans et al. 2014) experiments in biological research. Recently, the development of autoluminescent plants that can emit light without consumption of electricity and fossil fuels has received increasing attention. Kwak et al. (2017) reported a nanobiotic approach to produce light-emitting plants. These authors introduced nanoparticles, respectively loading luciferase, luciferin, and coenzyme A to emit light, as well as semiconductor nanocrystal phosphors to shift the emission wavelength. In this research, plants were immersed in the nanoparticle solution and pressurized to intake the particles, resulting in 21.5 h of luminescence lifetime. However, it is more practical and sustainable if luminescence is initiated automatically without the addition of exogenous materials. Autoluminescence has been enabled by introducing complete biosynthetic pathways for luciferin and luciferase from the bacteria *P. leiognathi* (Krichevsky et al. 2010). Stronger autoluminescence was achieved by reconstituting the luciferin biosynthetic and recycling pathways from the mushroom *N. nambi* (Mitiouchkina et al. 2020), and by protein engineering and pathway optimization (Shakhova et al. 2024). In the mushroom, the luciferin (3-hydroxyhispidin) is synthesized from caffeic acid by two enzymes, and the oxyluciferin (caffeylpyruvic acid) is converted to caffeic acid by a single enzymatic step (Kotlobay et al. 2018). Given that all plants endogenously produce caffeic acid in the shikimate pathway, the hispidin-type luminescence pathway is highly compatible with plants. There are still many uncharacterized luciferin synthesis pathways in the world. It is expected that pathway elucidation and its application will lead to further improvement in the bioengineering of luminescent plants.

## Trends in synthetic biology in major countries

### a) USA

In 2000, seminal works on gene circuits resembling electronic transmitters and switches, utilizing transcriptional activity networks, were groundbreaking in synthetic biology (Elowitz and Leibler 2000; Gardner et al. 2000). Projects received substantial funding, including the National Science Foundation’s Synthetic Biology Engineering Research Center (SynBERC) program, based at the University of California, Berkeley. SynBERC facilitated collaboration between scientists from academia and industry, fostering basic technology development and researcher training. Its successor, the Engineering Biology Research Consortium (EBRC), was launched in 2016 (Si and Zhao 2016).

The EBRC outlined a research roadmap for the next-generation bioeconomy in 2019, critically evaluating the current state and potential of synthetic biology (https://ebrc.org/focus-areas/roadmapping/engineering-biology-2019/ (Accessed Jul 29, 2024)). The roadmap’s technical focus is “bottom-up”, emphasizing tool and technology innovations. Application and impact areas adopt a “top-down” approach, exploring the contributions of synthetic biology to national and global challenges. The technology themes include (1) DNA engineering, (2) molecular biotechnology, (3) host engineering, and (4) data science. The “Application and Impact” sectors include (1) biotechnology in industry, (2) health and medicine, (3) food and agriculture, (4) biotechnology in the environment, and (5) energy (Table 2).

**Table 2:** Table 2. Societal challenges and science/engineering aims.

Produce more food for a growing global population
Improve agricultural yields by increasing crop efficiency and production
Increase the availability and consistency of agricultural crop production by combating stressors and expanding consumable species
Improve the production and yield of meat from livestock and fish
Enable and advance the production and availability of non-vertebrate animal food sources
Improve production of “clean meat”
Advance the quality of plant-based meat products and improve large-scale manufacturing capabilities
Engineer microorganisms for nutrient production
Increase and improve the nutritional content and value of food
Increase the nutrient content in agricultural crops
Improve the healthiness of agricultural crops by enabling the reduction and elimination of toxins
Increase the nutrient content and value from animal food sources

The US Department of Energy’s Bioenergy Research Center (BRC) program aims to enhance plant functions to solve energy and environmental challenges (https://genomicscience.energy.gov/bioenergy-research-centers/ (Accessed Jul 29, 2024)). Objectives include ensuring energy security, reducing greenhouse gas emissions, expanding biobased product diversity, minimizing toxic chemical production, and creating rural job opportunities.

Within the Department of Defense, agencies such as the Defense Advanced Research Projects Agency (DARPA), the US Army, Navy, and Air Force lead synthetic biology research. Programs launched by DARPA, such as Living Foundries and Safe Genes, focus on engineering fundamental metabolic processes to produce complex molecules and enable scalable, adaptable molecule production (https://www.darpa.mil/program/living-foundries (Accessed Jul 29, 2024)).

### b) European Union and UK

Horizon 2020, spanning from 2014 to 2020, allocated €3.7 billion to the bioeconomy, emphasizing applied development, with funding from companies (https://cordis.europa.eu/projects/en (Accessed Jul 29, 2024)). Horizon Europe, operational from June 2021, offers €95.5 billion over seven years, with up to four missions targeting climate change and food-related challenges (https://research-and-innovation.ec.europa.eu/document/download/9224c3b4-f529-4b48-b21b-879c442002a2_en?filename=ec_rtd_he-investing-to-shape-our-future.pdf (Accessed Jul 29, 2024)).

MaxSynBio, a research consortium led by the Max Planck Institute, aims to construct artificial cells bottom-up (https://www.maxsynbio.mpg.de/13480/maxsynbio (Accessed Jul 29, 2024)). In systemic biotechnology at the Jülich Research Center, microbial metabolism and regulatory networks are studied for bioprocess development, alongside microalgae bioreactors (https://www.fz-juelich.de/en/institutes/ibg (Accessed Jul 29, 2024)). The Fraunhofer Institute for Interface Engineering and Biotechnology focuses on biorefinery technology using algae and food residues (https://www.ime.fraunhofer.de/en/Research_Divisions/business_fields_MB.html (Accessed Jul 29, 2024)). The Leibniz Institute for Agricultural Engineering and Bioeconomy explores biomass utilization for materials and energy (https://www.leibniz-gemeinschaft.de/en/institutes/leibniz-institutes-all-lists/leibniz-institute-foragricultural-engineering-and-bioeconomy (Accessed Jul 29, 2024)).

In France, “Genomic Medicine France 2025” and a bioeconomy strategy promote genomic healthcare and biomass usage (https://solidarites-sante.gouv.fr/IMG/pdf/genomic_medicine_france_2025.pdf (Accessed Jul 29, 2024); https://agriculture.gouv.fr/bioeconomy-strategy-france-2018-2020-action-plan (Accessed Jul 29, 2024)). A collaboration with the National University of Singapore on synthetic and systems biology was initiated in 2018 (https://www.inrae.fr/actualites/biologiesystemes-biologie-synthetique-bioeconomie-linra-cnrslinsa-toulouse-unissent-leurs-forces-asie-du-sud-est (Accessed Jul 29, 2024)).

The UK’s “Synthetic Biology for Growth” program, running from 2014 to 2022, invested £102 million to establish Synthetic Biology Research Centers (SBRCs), advance DNA synthesis technology, enhance training, and fund start-ups (https://www.ukri.org/what-we-do/browse-our-areas-of-investment-and-support/synthetic-biology-for-growth/ (Accessed Jul 29, 2024)). Centers at the University of Nottingham and the John Innes Center focus on biomass and plant nutritional fortification, respectively (https://www.ukri.org/what-we-do/browse-our-areas-of-investment-and-support/synthetic-biology/ (Accessed Jul 29, 2024)). The UK Research and Innovation (UKRI) website provides information on recently funded projects (https://www.ukri.org/whatwe-do/browse-our-areas-of-investment-and-support/synthetic-biology/ (Accessed Jul 29, 2024)).

### c) Australia

Australia’s National Science Agency presented “A National Synthetic Biology Roadmap” in 2021, guiding the nation’s trajectory (https://www.csiro.au/-/media/Services/Futures/Synthetic-Biology-Roadmap.pdf (Accessed Jul 29, 2024)). It positions synthetic biology as a transformative field ushering in novel manufacturing technologies (biomanufacturing) and engineered biological products. This roadmap highlights the ability of synthetic biology to swiftly develop functional DNA-encoded components and systems with enhanced predictability and precision compared with traditional genetic modification methods, thus offering value across various industries.

In May 2023, the Australian government issued the “List of Critical Technologies in the National Interest”, recognizing seven categories, including biotechnology, with synthetic biology as a specified domain (https://www.industry.gov.au/publications/list-critical-technologies-national-interest (Accessed Jul 29, 2024)). Australia’s comprehensive promotion of synthetic biology spans basic research, industrial growth, and national defense and security strategies.

The CSIRO allocated USD 13 million to the Synthetic Biology Future Science Platform (SynBio FSP) in 2016 to foster innovation and commercialization opportunities in manufacturing, industrial biotechnology, environmental remediation, agriculture, and healthcare (https://research.csiro.au/synthetic-biology-fsp/ (Accessed Jul 29, 2024)). The SynBio FSP encompasses six research areas: foundation technologies, industrial biotechnology, environment and biocontrol, health, and medicine, maximizing impact, and agriculture and food. These research areas focus on developing tools, advancing industries, managing the environment, enhancing health innovations, addressing social and ethical aspects, and agricultural innovation (https://www.csiro.au/en/research/production/biotechnology/Synthetic-Biology (Accessed Jul 29, 2024)). The initiative also hosts the CSIRO BioFoundry (https://www.csiro.au/en/work-with-us/use-our-labs-facilities/BioFoundry (Accessed Jul 29, 2024)), positioning Australia at the forefront of modern science advancement.

### d) China

In China, synthetic biology represents an evolving field with a broad spectrum of applications akin to its Western counterparts. Embraced within China’s future R&D plans, particularly outlined in the Chinese Academy of Sciences (CAS) roadmap, synthetic biology is anticipated to not only deepen our understanding of fundamental biological mechanisms but also unlock vast practical applications (Pei et al. 2011). According to the CAS, synthetic biology encompasses two primary facets: the design of novel biological components and systems, and the redesign of existing biological systems. This approach aims to elucidate natural mechanisms and systems, while harnessing them for human benefit (http://www.bcas.cas.cn/sr/mega/202003/t20200318_231548.html (Accessed Jul 29, 2024)).

Synthetic biology initiatives in China, such as the “Synthetic Cell Factory Project”, align with national strategic plans, including the National Key Basic Research Development Plan (973 Plan), laying crucial groundwork for the advancement of synthetic biology (Table 3; Zhang 2019). Under the auspices of the CAS, institutes such as the Beijing Institute of Genomics, Qingdao Institute of Bioenergy and Bioprocess Technology, and Tianjin Institute of Industrial Biotechnology have spearheaded pivotal research endeavors (https://english.big.cas.cn (Accessed Jul 29, 2024); http://english.qibebt.cas.cn (Accessed Jul 29, 2024); https://english.tib.cas.cn (Accessed Jul 29, 2024)).

**Table 3:** Table 3. Synthetic biology research projects supported by the “National Key Basic Research Program” (973 program) in China (2011–2019).

Project Title	Year
Synthetic cell factory	2011–2015
Photosynthesis and artificial photosynthesis leaves	2011–2015
Construction and integration of new functional artificial biodevices	2012–2016
Artificial synthesis system that realizes innovation and superior production of microbial medicines	2012–2016
Building new routes to synthesize bio-based materials using synthetic biology techniques	2012–2016
Structure and mechanism of stress-resistant parts	2013–2017
Suitability of synthetic microbial systems	2013–2017
Design and synthesis of microbial multicellular systems	2014–2018
Basic research on synthetic biological devices for bladder cancer	2014–2018
Artificial design and system optimization of biological nitrogen fixation and related stress tolerance modules	2015–2019

Chinese engagement in international synthetic biology initiatives is underscored by events such as the regional rounds of the “International Genetically Engineered Machine Contest” (iGEM) held in Hong Kong from 2011 to 2013 (https://2011.igem.org/Regions/Asia/Jamboree (Accessed Jul 29, 2024)). Furthermore, the establishment of a synthetic biology laboratory at the Shenzhen Institute of Advanced Technology in 2019 signifies China’s commitment to fostering cutting-edge research in this domain.

In China’s “Outline of the 14th Five-Year Plan and Long-Term Goals for 2035”, genetic biotechnology emerges as a pivotal focus area alongside AI and quantum technology. The plan emphasizes genomics research, cell engineering, molecular breeding, synthetic biology, and biopharmaceutical innovation, among other priorities (http://www.gov.cn/xinwen/2021-03/13/content_5592681.htm (Accessed Jul 29, 2024)). Moreover, the “14th Five-Year Plan for the Development of Bioeconomy” articulated by the National Development and Reform Commission underscores China’s ambition to lead the global bioeconomy by 2035, emphasizing technological prowess, industrial integration, biosecurity, and institutional R&D systems (Zhang X et al. 2022).

### e) Japan

In Japan, synthetic biology, as outlined by the Center for Research Development Strategies in its 2021 report on Life Science and Clinical Medicine, encompasses two primary approaches: top-down and bottom-up. The former involves cell redesign through genome editing, whereas the latter focuses on constructing artificial biomacromolecules or molecular systems with cell-like functions using biomolecules or modified molecules. Synthetic biology combines a fundamental aspect of creating “possible life” with an engineering and medical aspect aimed at developing useful molecules and life systems leveraging advanced production methods. While the top-down approach is industrializing, particularly in fermentation and metabolic engineering, the bottom-up approach remains largely scientific.

Japan initiated the “Bio Strategy 2019” in response to the need for a national biological field strategy after a decade-long gap. Updated in 2020 to address post-pandemic economic recovery and climate change, this strategy signals a significant shift in Japan’s synthetic biology research direction. The Cabinet’s approval of the “Biostrategy Follow-up” underscores the commitment to adapt to evolving trends, including ambitious targets for greenhouse gas emissions reduction.

In material production leveraging biological functions, Japan focuses on initiatives such as the DBTL cycle for gene discovery and productivity enhancement. With a heritage in enzyme-based fermentation, synthetic biology is often viewed as an extension of metabolic engineering. Projects by the New Energy and Industrial Technology Development Organization (NEDO) aim to produce pharmaceuticals and chemicals using smart cells. The Japan Science and Technology Agency (JST) has funded 19 research projects (2008–2020) under its Strategic Creative Research Promotion Program, including genome design and synthesis endeavors. The project’s website states, “While research and development using long DNA is accelerating worldwide, and several centers have been established in the United States, China, and the United Kingdom to strategically invest in basic research, technology development, and start-up company fostering, there appears to be little R&D on design guidelines for a genome that can control cells at will”. Some themes show originality, such as attempts to control proliferation and division for utilizing artificial cells as reactors (https://www.jst.go.jp/kisoken/crest/en/research_area/ongoing/areah30-1.html (Accessed Jul 29, 2024)). In addition to the production of useful substances through genetic modification, the development and production of agricultural and fishery products using genome editing technology have also been put to practical use (Matsuo and Tachikawa 2022).

## Future directions and perspectives

Synthetic biology finds extensive applications across academic and industrial sectors, including the innovation of food, biofuels, pharmaceuticals, and bioremediation. Microbial-based precision fermentation, in particular, has emerged as a potent tool, addressing food security challenges while offering environmentally sustainable high-quality food production (Shi et al. 2022). In recent years, plant synthetic biology has garnered attention for diverse applications, such as metabolite production, crop breeding, biosensors, and stress tolerance enhancement, employing a constitutive approach (Gupta et al. 2021). Stress response mechanisms in plants involve intricate regulatory networks comprising sensor proteins, enzymes, transcription factors, epigenetic regulators, and post-translational modifiers. While genetic engineering interventions targeting individual components have shown promise, the future necessitates the rational design of stress-responsive genetic circuits rooted in synthetic biology principles (Lohani et al. 2022).

Plants serve as hosts for the production of secondary metabolites utilized in pharmaceuticals, spices, dyes, and fragrances. Leveraging plant metabolic systems for substance production in microorganisms will advance with innovative technologies such as genome editing, AI design, and high-throughput sequencing. Plants offer advantages, such as efficient expression of plant-derived enzymes, storage of compounds toxic to microorganisms, and intricate cell organelles enabling precise metabolic pathway manipulation. Plant-based bioproduction offers sustainability through low-cost synthesis of various valuable natural products via photosynthesis. Energy crop development mitigates land and food crop competition, facilitating further plant cell factory expansion. However, the complex metabolic control systems in plants pose challenges to predictable productivity. Addressing this requires comprehensive investigation of plant endogenous metabolism, practical planning, and establishment of tissue culture techniques (Liu et al. 2023; Zhu et al. 2021).

Successful examples in plant-based material production through synthetic biology represent only a fraction of the potential. Continued technological advancements are expected to drive the social integration of plant-based material production and basic science via synthetic biology methodologies.
